# Rapid and reversible suppression of ALT by DAXX in osteosarcoma cells

**DOI:** 10.1038/s41598-019-41058-8

**Published:** 2019-03-14

**Authors:** Kathryn E. Yost, Sarah F. Clatterbuck Soper, Robert L. Walker, Marbin A. Pineda, Yuelin J. Zhu, Corbin D. Ester, Soyeon Showman, Anna V. Roschke, Joshua J. Waterfall, Paul S. Meltzer

**Affiliations:** 1 Genetics Branch, Center for Cancer Research, National Cancer Institute, National Institutes of Health, Bethesda, MD 20892 USA; 2 Present Address: Center for Personal Dynamic Regulomes, Stanford University School of Medicine, Stanford, CA 94305 USA; 3 Present Address: Harvard Medical School, Boston, MA 02115 USA; 4 Present Address: Translational Research Department & INSERM U830, Institut Curie, Paris, France

## Abstract

Many tumors maintain chromosome-ends through a telomerase-independent, DNA-templated mechanism called alternative lengthening of telomeres (ALT). While ALT occurs in only a subset of tumors, it is strongly associated with mutations in the genes *ATRX* and *DAXX*, which encode components of an H3.3 histone chaperone complex. The role of *ATRX* and *DAXX* mutations in potentiating the mechanism of ALT remains incompletely understood. Here we characterize an osteosarcoma cell line, G292, with wild-type *ATRX* but a unique chromosome translocation resulting in loss of DAXX function. While ATRX and DAXX form a complex in G292, this complex fails to localize to nuclear PML bodies. We demonstrate that introduction of wild type *DAXX* suppresses the ALT phenotype and restores the localization of ATRX/DAXX to PML bodies. Using an inducible system, we show that ALT-associated PML bodies are disrupted rapidly following DAXX induction and that ALT is again restored following withdrawal of DAXX.

## Introduction

The unlimited proliferative capacity of cancer cells is closely linked to maintenance of telomeres. Active mechanisms must both counteract the shortening of telomeres with each cell cycle (the end-replication problem) and prevent telomere ends from inducing double strand break DNA damage repair pathways (the end-protection problem). Cancer cells maintain telomeres using at least two distinct mechanisms: reactivation of telomerase and the alternative lengthening of telomeres (ALT) pathway^1,2^. In ALT, telomeres are lengthened through DNA-templated DNA synthesis, in a mechanism that relies upon homologous recombination machinery. Hallmarks of ALT include heterogeneous telomere length, the presence of extrachromosomal telomere repeats (ECTR), and localization of telomere sequences to nuclear PML bodies, forming ALT associated PML bodies (APBs)^3,4^. ALT is observed in 10–15% of tumors overall^5^, but its frequency is histotype specific. In osteosarcoma, the most common form of primary bone cancer, the prevalence of ALT is roughly 35%^6^.

ALT is strongly associated with somatic loss of function mutations in *ATRX* and *DAXX*^7^. ATRX and DAXX form a histone chaperone complex responsible for deposition of non-canonical histone variant H3.3 at heterochromatic regions of the genome including pericentromeric and telomeric regions^8–10^. Depletion of ATRX does not immediately trigger ALT in fibroblasts, but does result in post-crisis ALT that can be suppressed by restoration of ATRX expression^11^. Similarly, depletion of ATRX or DAXX in the context of induced telomere damage appears sufficient to induce an ALT-like phenotype^12^. Because ATRX and DAXX each have multiple functions in the cell, it has remained unclear precisely how these proteins act to suppress ALT. In addition to its role chaperoning H3.3, ATRX has been implicated in resolution of G-quadruplex structures at telomeres^13^ and resolution of telomere cohesion prior to mitosis^14^, thus preventing telomere breakage and preserving genome integrity. Additionally, reintroduction of ATRX is sufficient to suppress ALT in an ATRX-null background^13^. Evidence suggests that ALT-associated DNA synthesis occurs downstream of DNA double-stranded breaks at telomeres^15^, so it is parsimonious to infer that these actions of ATRX at telomeres prevent the occurrence of breaks that underpin ALT.

When complexed with ATRX, DAXX directly binds H3.3 and is required for its deposition at telomeric regions, but whether this is the sole function responsible for DAXX suppression of ALT remains unclear^10^. ALT-associated *DAXX* mutations are particularly frequent in pancreatic neuroendocrine tumors (22%^16^), but have also been reported in several other cancer types including adrenocortical carcinoma^17^ and pediatric glioma^18^. In general, the *DAXX* mutation frequency is lower than that of *ATRX*, but these relative frequencies may reflect the size of the mutational target. Although recurrent *ATRX* mutations are found in osteosarcoma tumors, alterations in *DAXX* have only recently been reported^19–21^.

We here describe characterization of a translocation disrupting *DAXX* in the osteosarcoma cell line G292. We characterize the rearranged *DAXX* gene and provide evidence that the DAXX fusion protein produced retains its ability to bind ATRX but fails to localize to PML bodies. We show that a short, 19 amino acid C-terminal sequence including the SUMO interaction motif (SIM) in DAXX is required for correct localization of both DAXX and ATRX. Upon reintroduction of full length *DAXX* in G292, telomere maintenance by ALT is rapidly suppressed, resulting in loss of C-circles and ALT-associated PML bodies (APBs), demonstrating that continued DAXX deficiency is essential to maintain ALT in this system. We generate G292-iDAXX, a cell line with inducible DAXX expression, and show that short-term expression of DAXX in G292 begins to disrupt APBs within hours and that this process is reversible upon withdrawal of DAXX. We conclude that DAXX is required for correct ATRX localization and suggest that G292-iDAXX is a useful model system for study of the ALT mechanism.

## Results

### G292 is an ALT cell line with a DAXX fusion

**Figure 1:**
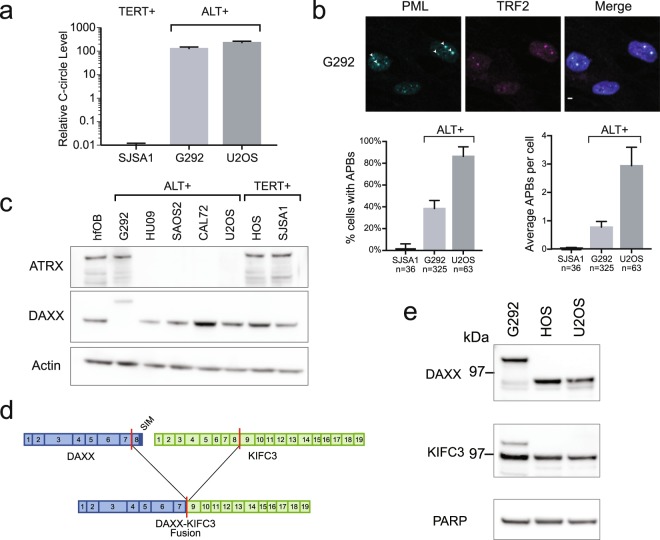
G292 is an ALT cell line with a DAXX fusion. (**a**) qPCR quantification of C-circle ECTR^40^. G292 C-circle ECTR were high as compared with TERT expressing osteosarcoma cell line SJSA1. G292 C-circle levels were comparable to ALT + osteosarcoma line U2OS. (**b**) APBs quantification using immunofluorescence for PML and TRF2. In images, arrowheads mark APBs, identified by colocalization of PML and TRF2. The scale bar represents 2 µM. As quantification reveals, APBs were common in G292 cells as in ALT line U2OS, but were infrequently observed in a TERT expressing osteosarcoma line. (**c**) Western blot for ATRX and DAXX proteins in a panel of osteosarcoma cell lines. Most ALT lines do not express ATRX, but G292 expressed ATRX at levels similar to TERT + osteosarcoma cell lines. DAXX was observed to have aberrant migration. All images are from the same gel, with cropped images of ATRX and DAXX bands from top half of gel (stripped and reprobed for DAXX), and actin bands from bottom half. Complete images in Supplementary Fig. S1b. (**d**) Depiction of the DAXX-KIFC3 fusion observed in G292 RNA-Seq. The fusion event results in the loss of Exon 8 of *DAXX*, encoding the final 19 amino acids of the DAXX protein, which contain a sumo-interacting motif (SIM) domain (dark blue). (**e**) Western blot of DAXX and KIFC3. In G292, DAXX-KIFC3 fusion was expressed at levels similar to DAXX in other osteosarcoma cell lines. Wild-type DAXX was not expressed in G292, but wild-type KIFC3 was observed. Expected molecular weights were 81 kDa for DAXX and 126 kDa for DAXX-KIFC3 fusion. All cropped images are from the same gel, which was stripped and reprobed. Complete images in Supplementary Fig. S1d.

The G292 osteosarcoma cell line was previously observed to rely on ALT for telomere maintenance^1^, though unlike the majority of ALT cell lines, it expresses wild-type ATRX^19^. Consistent with previous reports, we observed hallmarks of ALT in G292 including high levels of C-circle extrachromosomal telomere repeats (ECTR) and APBs (Fig. 1a,b), as well as high telomere repeat content (Supplementary Fig. S1). We confirmed protein expression of ATRX and DAXX in G292 by western blot, revealing normal ATRX protein expression but aberrant size of DAXX protein in comparison with other osteosarcoma cell lines (Fig. 1c). This observation suggested a potential fusion event involving *DAXX*. Analysis of the G292 transcriptome by RNA-seq identified a novel *DAXX* translocation that results in an in-frame fusion transcript spliced from *DAXX* exon 7 to exon 9 of *KIFC3* (Fig. 1d). The *DAXX-KIFC3* fusion was verified by RT-PCR (Supplementary Fig. S1), and the predicted 126 kDa product was confirmed by western blot using both DAXX and KIFC3 antibodies (Fig. 1e). No wild-type *DAXX* transcript was detected by either RNA-Seq or RT-PCR in G292 (Supplementary Fig. S1). While this report was in preparation, Mason-Osann *et al*. reported a *DAXX-KIFC3* fusion in G292 consistent with that described here^21^.

### Loss of C-terminal DAXX sequence drives mislocalization of DAXX-KIFC3 and ATRX

**Figure 2:**
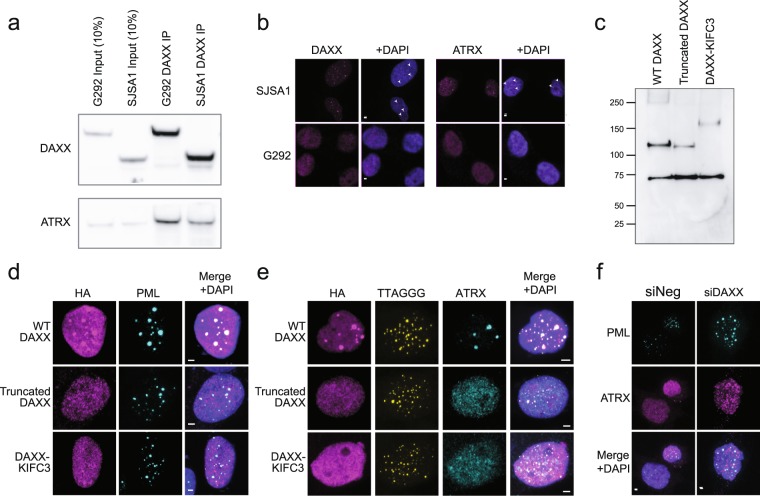
Loss of C-terminal DAXX sequence drives mislocalization of DAXX-KIFC3 and ATRX. (**a**) Western blot of DAXX immunoprecipitation. DAXX-KIFC3 was competent to bind ATRX in G292, similar to wild-type DAXX in SJSA1 cells. All cropped images are from the same gel, which was stripped and reprobed. Complete images in Supplementary Fig. S2a. (**b**) Immunofluorescence imaging showing localization of DAXX and ATRX in ALT + and TERT + osteosarcoma cell lines. In SJSA1 osteosarcoma cells wild-type DAXX and ATRX were localized to nuclear punctae (arrowheads), but in G292 the proteins were observed to be diffusely localized throughout the nucleus. Scale bars represent 2 µm. (**c**) Western blot showing expression of HA-tagged wild-type DAXX, truncated DAXX and DAXX-KIFC3 in G292. Expected MW were 83 kDa for the HA-tagged truncated DAXX and 129 kDa for HA-tagged DAXX-KIFC3. The band at 70 kDa was non-specific. Blank lanes have been cropped. **(d)** Immunofluorescence imaging of transiently expressed WT DAXX, truncated DAXX and DAXX-KIFC3 in G292 cells. Exogenous WT DAXX was enriched at PML bodies unlike either truncated DAXX or exogenous DAXX-KIFC3. Scale bars represent 2 µm. (**e**) Immunofluorescence imaging showing localization of ATRX with transiently expressed WT DAXX, truncated DAXX and DAXX-KIFC3 in G292 cells. ATRX forms nuclear foci colocalizing with wild-type DAXX but remains diffuse in cells expressing exogenous truncated DAXX or the DAXX-KIFC3 fusion. Scale bars represent 2 µm. (**f**) Immunofluorescence imaging showing localization of ATRX in the absence of DAXX-KIFC3. Knock-down of DAXX-KIFC3 (Supplementary Fig S2b) did not restore ATRX localization to PML bodies. Scale bars represent 2 µm.

ATRX and DAXX form a complex to chaperone deposition of histone variant H3.3 at pericentric and telomeric heterochromatin. To assess the impact of the DAXX-KIFC3 fusion on the function of this complex, we asked whether the fusion protein could still interact with ATRX. Using immunoprecipitation with a DAXX antibody, we found that DAXX-KIFC3 still interacts with ATRX in G292 (Fig. 2a). The DAXX fusion protein was also reported to co-immunoprecipitate H3.3^21^. Typically, ATRX and DAXX localize with H3.3 to PML bodies^22^. Using immunofluorescence imaging, we assayed the localization of ATRX and DAXX in G292 and the TERT + osteosarcoma line SJSA1. We observed that both ATRX and DAXX have a punctate nuclear staining in SJSA1 consistent with localization at PML bodies^19^ but are diffuse throughout the nucleus in G292, suggesting that the DAXX-KIFC3 fusion cannot properly localize to PML bodies (Fig. 2b).

Previous reports have shown that DAXX contains a C-terminal SUMO Interacting Motif (SIM) domain responsible for its localization to nuclear PML bodies^23^. This SIM domain is lost in the DAXX-KIFC3 fusion protein, so we anticipated that the loss of this sequence was responsible for the failure of DAXX and ATRX to be enriched at PML bodies in G292. To test this hypothesis, we generated HA tagged versions of wild-type DAXX, DAXX-KIFC3, and DAXX truncated at the fusion breakpoint and lacking the SIM domain (Fig. 2c). We transiently transfected G292 cells with these constructs and assayed localization with an antibody against the HA tag. We found that only wild-type full-length DAXX localized correctly to nuclear PML bodies (Fig. 2d). Similarly, ATRX co-localized in nuclear foci with wild-type DAXX but remained diffuse in cells expressing truncated DAXX or exogenous DAXX-KIFC3 (Fig. 2e). These results directly demonstrate that failure of DAXX-KIFC3 to localize in G292 is due to the loss of its C-terminal SIM domain and does not result from an effect of the conjoined KIFC3 sequence. Furthermore, this result also confirms that ATRX localization to PML bodies depends on DAXX in a C-terminal SIM domain dependent manner.

To test whether DAXX-KIFC3 might represent a gain-of-function, we used siRNA to knock down its expression. Knockdown of DAXX-KIFC3 does not result in a change in growth rate of G292 cells (Supplementary Fig S2). Furthermore, loss of DAXX-KIFC3 does not relieve the ATRX localization defect in G292 (Fig. 2f). Thus, the DAXX-KIFC3 fusion in G292 disrupts DAXX function resulting in failure of both ATRX and DAXX to correctly localize to nuclear PML bodies.

### Stable expression of wild-type DAXX suppresses ALT in G292

**Figure 3:**
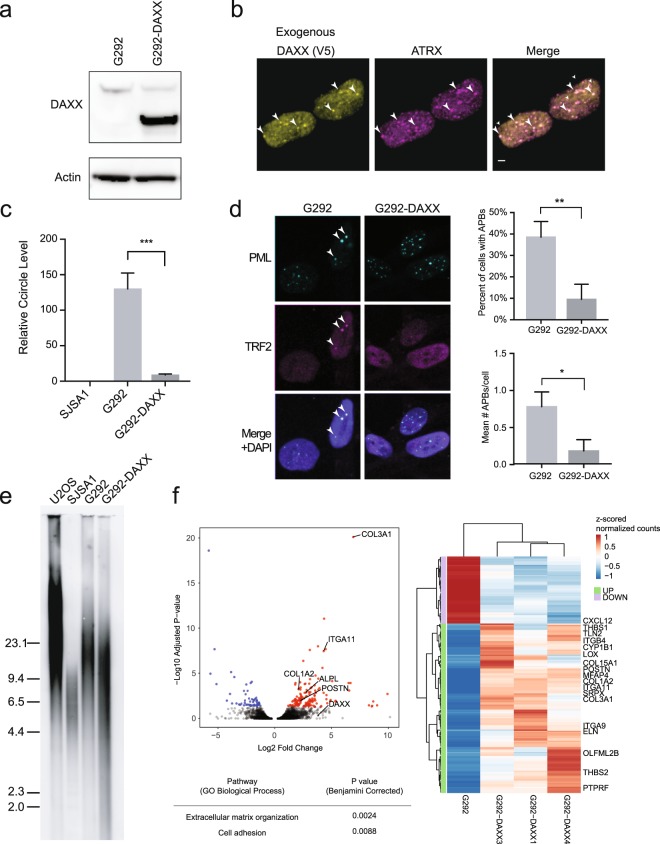
Stable expression of wild-type DAXX abrogates ALT, leading to telomere erosion and a differentiation signature. (**a**) Immunoblot using an antibody against DAXX showing stable reintroduction of DAXX in G292. All cropped images are from the same gel, which was stripped and reprobed. Complete images in Supplementary Fig. S3a. (**b**) Immunofluorescence against V5-tagged DAXX and ATRX showing relocalization of ATRX in G292-DAXX cells. Arrowheads highlight colocalization of DAXX and ATRX foci. Scale bars represent 2 µm. (**c**) qPCR quantification for C-circle ECTR. C-circles were eliminated in G292-DAXX cells (unpaired t-test, p < 0.001). (**d**) Immunofluorescence imaging for quantification of APBs using antibodies against TRF2 and PML. Sample images show APBs in parental G292 (arrowheads), but not G292-DAXX. Quantification reveals that APBs were significantly reduced in G292-DAXX, in both percent of cells with APBs (unpaired t-test, p < 0.01) and mean APBs per cell (unpaired t-test, p < 0.05). (**e**) Telomere restriction fragment Southern blot showing erosion of telomeres in G292-DAXX after 20 passages in culture. The extent of telomere shortening observed in G292-DAXX was consistent with the erosion of telomeres due to the end-replication problem (estimated 50–200 bp lost per cell division^41^). Blank lanes were cropped. (**f**) Volcano plot comparing three independent clones of G292-DAXX to the parental G292 cell line reveals significant upregulation of genes associated with osteoblast differentiation. Upregulated genes are significantly enriched for ECM organization and cell adhesion GO ontology terms, which are highlighted in the heatmap showing changes in expression of significantly altered genes. No ontology terms were significantly associated with downregulated genes.

To test the role of DAXX-KIFC3 in the ALT status of G292, we generated a cell line, G292-DAXX, stably expressing V5-tagged wild-type DAXX (Fig. 3a). We observed that V5-tagged DAXX localized with ATRX to punctae in the nucleus (Fig. 3b) of G292-DAXX cells. In the G292-DAXX cell line, we observed significant reductions in multiple hallmarks of ALT, including a fourteen-fold decrease in C-circles (*p* = 0.0007, unpaired t-test; Fig. 3c) and a four-fold decrease in the both the percentage of cells with APBs (*p* = 0.077, unpaired t-test) and average number of APBs per cell (*p* = 0.014, unpaired t-test; Fig. 3d), demonstrating that ongoing abrogation of DAXX function is necessary for maintenance of ALT in G292.

We next assessed the long-term effects of ALT suppression in G292-DAXX cells. Using the TRAP assay, we ascertained that telomerase activation did not occur upon suppression of ALT (Supplementary Fig. S3), demonstrating that these cells lack both the ALT and TERT mechanisms of telomere maintenance. We speculate that this lack of telomere maintenance contributed to the observed slow growth of this cell line (Supplementary Fig. S3). After 20 passages, telomere length of G292-DAXX was assayed by Southern blot, demonstrating erosion of telomeres relative to the parental cell line consistent with the lack of a telomere maintenance mechanism (Fig. 3e).

To further reveal effects of wild-type DAXX expression in G292, we performed RNA-Seq of the constitutive G292-DAXX cell lines. Pathway analysis identified significantly upregulated pathways related to osteoblast differentiation, including extracellular matrix organization (*p* = 0.0024) and cell adhesion (*p* = 0.0088), illustrated by upregulation of collagen genes and the bone mineralization enzyme alkaline phosphatase (Fig. 3f). Notably, expression of genes identified as targets of DAXX repression^24^ was not significantly altered in the G292-DAXX cell lines (Supplementary Fig. S3). We confirmed the differentiation phenotype with alkaline phosphatase staining, which shows higher baseline staining in the G292-DAXX cells compared to empty vector controls, and more rapid increase of alkaline phosphatase activity following growth in osteoblast differentiation media (Supplementary Fig. S3). Taken together, we conclude that expression of wild-type DAXX and long-term loss of telomere maintenance result in reduced proliferation and increased expression of bone differentiation genes.

### Short-term DAXX expression rapidly suppresses ALT in G292

**Figure 4:**
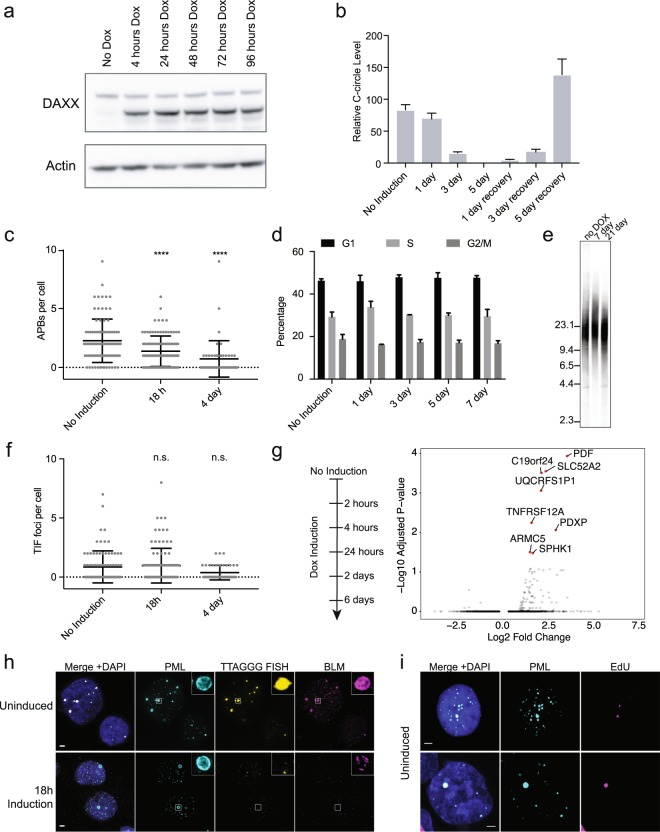
Inducible expression of wild-type DAXX in G292 abrogates ALT with rapid kinetics. (**a**) Western blot with anti-DAXX antibody showing kinetics of DAXX induction in the G292-iDAXX cell line. DAXX was induced using 10 ng/mL doxycycline. All cropped images are from the same gel, which was stripped and reprobed. Complete images in Supplementary Fig. S4a. (**b**) C-circles were assayed using the qPCR protocol. C-circles began to decrease immediately upon DAXX induction and were eliminated after 5 days of continuous DAXX expression. C-circles returned after DAXX induction was stopped, showing that shutdown of ALT is reversible in this cell line. (**c**) Quantification of APBs using TTAGGG FISH and PML immunofluorescence in G292-iDAXX. Colocation was quantified using previously reported tools^13^. A significant reduction in APBs was observed as soon as 18 h after DAXX induction (ANOVA multiple comparisons test *p* < 0.001), samples images in Supplementary Fig. S4b. (**d**) FACS cell cycle analysis using propidium iodide showed no changes in cell cycle progression after a week of continuous DAXX expression in G292-iDAXX (*p* = 0.92, 2-way ANOVA). Plots represent the mean of triplicate cultures. Sample FACS traces and fits are found in Supplementary Fig. S4c. (**e**) Telomere length Southern blot showed undetectable levels of telomere erosion after DAXX expression of up to three weeks. Blank lanes were cropped. (**f**) Quantification of TIFs using TTAGGG FISH and 53BP1 immunofluorescence in G292-iDAXX. Colocation was quantified as for APBs. TIFs per cell were not significantly reduced after 4 days of DAXX expression (*p* = 0.95 at 18 h, *p* = 0.09 at 4d; ANOVA multiple comparisons test), samples images in Supplementary Fig. S4b. (**g**) Volcano plot of comparing RNA-seq data from short-term DAXX induction for the indicated time points compared to uninduced cells reveals minimal consistent changes in gene expression. (**h**) Structured illumination imaging of immunofluorescence plus TTAGGG FISH in G292-iDAXX. Large APBs including strong telomere FISH signal and BLM helicase were common in the ALT state but appeared to be lost rapidly after DAXX induction. Contrast was increased for 18 h induction insets to reveal details. Scale bars represent 2 µm. (**i**) PML IF plus EdU imaging demonstrated that APBs are a site of new DNA synthesis in the ALT state. EdU is incorporated at the site of new DNA synthesis. In G292-iDAXX cells in the ALT state, EdU is incorporated at PML bodies, even outside of S-phase. Scale bars represent 2 µm.

To further explore the immediate effects of DAXX expression in G292, and to circumvent issues attributable to long-term deficits in telomere maintenance, we generated a cell line, G292-iDAXX, with doxycycline inducible DAXX expression (Fig. 4a). As in G292-DAXX, the expression of wild-type DAXX eliminated the ALT phenotype in G292-iDAXX. C-circles were dramatically decreased after three days of induction and were undetectable after five days of DAXX expression (Fig. 4b). Levels of APBs were also significantly decreased after only 18 hours of DAXX induction (*p* =< 0.0001 at 18 h and 4d, ANOVA multiple comparisons test; Fig. 4c). Notably, C-circle levels returned upon cessation of DAXX expression, though ALT recovery took several days. As C-circles are considered diagnostic for ALT^25^, this finding indicates that the shutdown of ALT is reversible in this system and that continuing DAXX deficiency is required for ALT activity. Despite suppression of the ALT phenotype, expression of wild-type DAXX for up to one week did not affect the cell cycle distribution in G292-iDAXX (*p* = 0.92, 2-way ANOVA) (Fig. 4d).

In U2OS, suppression of ALT by expression of wild-type ATRX is associated with rapid shortening of telomeres and a decrease in telomere dysfunction foci (TIFs)^13^. In G292-iDAXX, however, telomere erosion is undetectable by Southern blot after three weeks of DAXX expression (Fig. 4e). Though the number of TIFs per cell trends lower after four days of DAXX expression, the change is not statistically significant (*p* = 0.95 at 18 h, *p* = 0.09 at 4d, ANOVA multiple comparisons test; Fig. 4f), in contrast to reports that TIFs are significantly reduced after four days of ATRX expression in U2OS^13^.

To explore the transcriptional response to short-term ALT suppression, we performed RNA-Seq in G292-iDAXX cells during a time course of DAXX induction starting at 2 hours up to 6 days. Short term induction of DAXX resulted in minimal consistent changes in the gene expression profile of G292-iDAXX across time points compared to uninduced cells (Fig. 4g). Genes with differential expression were not enriched for specific biological processes based on gene ontology and do not serve known roles in telomere maintenance. Additionally, expression of neither canonical DAXX target genes nor the osteoblast differentiation signature genes were significantly altered following short-term induction of DAXX (data not shown). Taken together, these results suggest that short-term expression of DAXX in G292 eliminates ALT without extensive pleiotropic effects, and that long-term phenotypic changes outside of ALT inhibition are only observed following long-term expression of DAXX. Thus, G292-iDAXX represents a useful model for the exploration of the ALT mechanism.

As APBs are thought to be the site of the ALT mechanism, we performed Elyra structured illumination (SIM) super-resolution microscopy to monitor APBs after short periods of DAXX expression (Fig. 4h). Using SIM, APBs were observed in uninduced G292-iDAXX as atypically large PML foci, with PML protein creating an outer sphere that appeared to enclose telomere sequence. Consistent with previous reports^26^, there was an appearance of telomere ends clustering at the APB sphere as well as localization of BLM protein (Fig. 4h, Uninduced insets). As early as 18 hours post DAXX induction, large PML foci remained but no longer featured localization of telomere sequence. Telomere FISH signal appeared overall decreased in induced cells, and BLM protein appeared dispersed (Fig. 4h, 18 h induction). The rapid decrease in telomere FISH signal is consistent with a decrease in telomere sequence as measured via qPCR assay (Supplementary Fig. S4), but contrasts with the lack of erosion observed by Southern blot. Taken together these data imply that telomere FISH signal observed in APBs is primarily extra-chromosomal. These observations are particularly notable in light of the fact that visualization of EdU incorporation confirmed that new DNA synthesis takes place within enlarged PML foci in G292-iDAXX (Fig. 4i). The presence of newly synthesized DNA in APBs is consistent with APBs being the site of the ALT mechanism. EdU incorporation is observed at APBs in the absence of other DNA synthesis in the nucleus, suggesting a DNA synthesis mechanism outside of S-phase genome replication. Given the strong telomere FISH signal in APB foci (Fig. 4h, uninduced), we infer that this signal represents ECTR generation and/or ALT telomere repeat synthesis. Thus, rapid disassembly of APBs indicates that wild-type DAXX expression begins to suppress ALT within hours, and that suppression of ALT results in rapid loss of ECTR.

## Discussion

While mutations in *ATRX* and *DAXX* are both strongly associated with ALT, prior mechanistic studies have focused primarily on ATRX. DAXX is a multifunctional protein with roles in cell death pathways, H3.3 deposition, and transcription regulation^23,24,27–33^. We here describe a *DAXX* mutation in an ALT positive osteosarcoma cell line, G292, and demonstrate that maintenance of ALT is dependent on ongoing DAXX deficiency. We show that reintroduction of wild-type DAXX in an inducible G292-iDAXX cell line eliminates multiple hallmarks of ALT within days, that changes in APBs are detectable within hours, and that ALT suppression is reversible. We further demonstrate that ALT suppression in this mutant is driven by mislocalization of DAXX, ATRX and H3.3 due to precise loss of the C-terminal SIM motif in DAXX. An important observation is that effects on ALT are observed at short time points when effects on transcription profiles and cell cycle are minimal. This suggests that the DAXX dependent activities suppressing ALT are not mediated through expression or proliferation mechanisms and that G292-iDAXX is a powerful model system for the direct study of the ALT mechanism without significant confounding effects.

The DAXX-KIFC3 fusion observed in G292 truncates only the final exon of the DAXX mRNA, resulting in a protein product missing the final 19 amino acids. The fusion protein retains the binding sites for both ATRX and H3.3^34,35^, but lacks the C-terminal “IIVLSDSD” SIM motif that mediates the localization of DAXX to nuclear PML bodies. We find that in the absence of this motif, both DAXX and ATRX fail to be enriched at these nuclear structures. We expect that this failure of targeting could lead to a set of consequences at telomeres including failure to deposit H3.3, failure to resolve G-quadruplex structures, and failure to resolve mitotic telomere cohesion. Further study will reveal precisely how ATRX and DAXX localization are tied to the suppression of ALT.

Previous work has described restoration of wild-type ATRX in U2OS, an ATRX-null ALT osteosarcoma cell line^13^. Our observations differed from results reported in U2OS in two ways. First, we did not observe a significant decrease in TIFs after four days of DAXX expression, as was observed in U2OS after four days of ATRX expression. This may be explained by the fact that G292 cells have a longer doubling time than U2OS. After longer DAXX expression the trend in decreasing TIFs may have reached significance. A second difference between these models is that in U2OS, expression of wild-type ATRX resulted in drastic shortening of telomere length in as little as 17 days^13^. Even with long-term DAXX expression, we only observed modest erosion of telomere length in G292 (Fig. 3e), and no shortening was evident with 21 days of DAXX expression (Fig. 4e). The extent of telomere shortening in G292-DAXX is consistent with erosion due to the DNA end-replication problem. The extreme shortening in U2OS, however, is more consistent with telomere trimming, as observed with overexpression of the telomere-length control factor TZAP in that cell line^36^.

The G292-iDAXX model represents a useful system for studying the mechanisms by which DAXX suppresses ALT. Using G292-iDAXX, we were able to visualize changes in APBs after short-term DAXX induction. Surprisingly, after only 18 hours of DAXX expression striking changes in APBs were observed. Specifically, the extremely bright telomere sequence foci found in ALT APBs became dispersed, leaving only shells of PML protein. Given our evidence that telomeres do not rapidly shorten upon DAXX expression in G292-iDAXX, we conclude that these super-bright telomere repeat foci consist primarily of extra-chromosomal telomere repeats (ECTR). The rapid disappearance of ECTR foci could be explained by several mechanisms. The ECTR may either simply be dispersed, degraded via a mechanism that is inhibited in the ALT state but becomes engaged when ALT is suppressed, or degraded via an ongoing rapid turnover mechanism in the absence of new ECTR synthesis. Given our evidence of new DNA synthesis at APBs (Fig. 4i), we favor the final interpretation, and speculate that in the ALT state ECTR are synthesized at a rate outstripping the capacity to degrade them. We conclude that dissolution of APBs indicates that wild-type DAXX rapidly leads to disruption of the site of the ALT mechanism.

Active telomere maintenance is an essential function of cancer cells. From a translational perspective it is particularly significant that suppression of ALT through re-introduction of WT DAXX was not associated with telomerase re-expression. In the stable cell lines, the long-term sequelae of this apparent lack of any telomere maintenance mechanism were telomere attrition, an increased differentiation phenotype, and significant cell proliferation defects. These results strongly support the ALT pathway as an attractive target for therapeutic intervention in ALT + tumors. We expect that further mechanistic insights into the pathways required to maintain ALT may identify essential steps amenable to therapeutic targeting. In particular, the short time scale in which changes to APBs can be observed in the G292-iDAXX system is amenable to many experimental and screening approaches and suggests the involvement of post-translational mechanisms potentially susceptible to small molecule inhibitors.

## Methods

### Cell Culture

Cell lines were obtained from ATCC. All cell line identities were verified by SNP genotyping and tested negative for mycoplasma. G292, HU09, CAL72, HOS, and SJSA-1 cell lines were maintained in RPMI media supplemented with 10% fetal bovine serum and penicillin/streptomycin. SAOS2 and U20S cell lines were maintained in McCoy’s 5A modified media supplemented with 15% fetal bovine serum and penicillin/streptomycin. The hFOB cell line was maintained in GMEM/F-12 media supplemented with 10% fetal bovine serum, 0.3 mg/mL G418 and penicillin/streptomycin and incubated at 34 °C.

### C-Circle Assay

C-circle levels were quantified using the quantitative PCR-based C-circle assay as described with the following modifications^37^. Genomic DNA was extracted using the DNeasy Blood and Tissue kit (Qiagen) quantified with a Qubit Fluorometer (Invitrogen). DNA was digested with 4 U/ug HinfI and AluI and 25 ng/ug RNase A. 16 ng of genomic DNA was added to 10 μL reaction containing 0.2 mg/mL BSA, 0.1% Tween-20, 4 mM DTT, 1 mM dNTPs, 1X phi29 buffer and 7.5 U phi29 (NEB) and incubated at 30 C for 8 hours then 65 C for 20 minutes. 10 μL of each C-circle reaction product were diluted with 30 μL of Tris-EDTA (10 mM Tris, 0.1 mM EDTA, pH 7.6). Each qPCR reaction contained 5 μL of diluted C-circle product (corresponding to 2 ng of input DNA), 1X Power SYBR Green PCR Master Mix (Life Technologies) and primer sets. Telomere primers generating a short, fixed length product were used^38^. VAV2 was used as the single copy gene reference gene. Final primer concentrations were 900 nM each for telomere forward and reverse primers and 700 nM/400 nM for VAV2 forward and reverse primers, respectively. PCR reactions were performed on the ABI 7900 Real Time PCR System with the following conditions: 95 °C for 15 min, 2 cycles of 94 °C for 15 seconds and 49 °C for 15 seconds, followed by 40 cycles of 95 °C for 15 seconds, 62 °C for 30 seconds and 74 °C for 1 minute.

### Immunofluorescence

Cells were grown either in optically clear bottom 24-well plates or on acid-washed coverslips and were fixed with 4% paraformaldehyde for 15–20 minutes at room temperature. Following three washes with PBS, cells were permeabilized with 1% Triton X-100 for 10 minutes at room temperature, washed three times with PBS and blocked with 20% donkey serum for one hour at room temperature. Incubation with primary antibodies diluted in 5% donkey serum was carried out at 4 °C overnight. Primary antibodies used included: rabbit polyclonal anti-ATRX (Santa Cruz, H-300, 1:200 dilution), rabbit polyclonal anti-DAXX (Sigma-Aldrich, HPA001906, 1:100 dilution), mouse monoclonal anti-PML (Santa Cruz, PG-M3, 1:200 dilution), rabbit polyclonal anti-TRF2 (Bethyl, A300, 1:200 dilution), mouse monoclonal anti-V5 (Thermo Fisher, R960, 1:400 dilution), rabbit polyclonal anti-HA (Santa Cruz sc-805, 1:250 dilution), rabbit polyclonal anti-53BP1 (Novus NB-100–904, 1:200 dilution). Following primary antibody incubation, cells were washed three times with PBS and incubated with Alexa Fluor conjugated secondary antibodies (Jackson ImmunoResearch or ThermoFisher, 1:400 dilution) diluted in 5% donkey serum for one hour at room temperature. Cells were washed three times with PBS and mounted in mounting media with DAPI.

### Immunofluorescence + Telomere FISH

For IF + FISH, cells were grown on acid-washed coverslips. IF was performed as above, with the modification that after permeabilization coverslips were treated with 100 µg/mL RNase A in PBS for 20–30 min at 37 °C followed by thorough rinsing with PBS. After the wash steps following the secondary antibody incubation, coverslips were re-fixed for 15 minutes in freshly made 4% paraformaldehyde in PBS at room temperature, then rinsed well with PBS and dehydrated. The TelC-Cy3 PNA FISH probe (Panagene) was diluted to 200 nM in hybridization buffer (70% deionized formamide, 10 mM Tris (pH 7.4), 0.5% 10x Roche blocking solution) and denatured at 90 °C for 5 minutes. Meanwhile, coverslips were denatured at 85 °C for 5 minutes. Coverslips were inverted onto 75 µL droplets of diluted probe and heated to 85 °C for an additional 5 minutes. Hybridization proceeded overnight at room temperature. Coverslips were then washed and mounted with Prolong Diamond mounting media with DAPI.

### Microscopy

All images were captured on a Zeiss LSM 780 confocal microscope using a 63x oil immersion objective. For Fig. 4h, Zeiss ELYRA structured illumination microscopy was used. Zeiss Zen Black software was used for microscope control and for processing of ELYRA images. Images were cropped, adjusted for brightness and contrast, and pseudo-colored using ImageJ. No non-linear adjustments were used.

### APB and TIF quantification

For quantification of APBs in parental or G292-DAXX cell lines, immunofluorescence for PML and TRF2 was performed as above following methionine restriction for four days. APBs were scored programmatically using ImageJ, with verification by visual inspection. For quantification of APBs in G292-iDAXX, methionine restriction was not used, and APBs were detected using PML IF plus telomere FISH. TIFs were scored as for APBs, using 53BP1 IF plus telomere FISH. APBs and TIFs were scored as colocalized foci as previously reported^13^.

### Immunoblotting

Immunoblotting was performed using standard procedures. Whole cell extracts were used except for the DAXX, KIFC3 and PARP blots in Fig. 1e. Briefly, cells were lysed in RIPA buffer supplemented with protease inhibitors, sonicated, and spun down at 12,500 rpm for 15 minutes at 4 °C. For the DAXX, KIFC3 and PARP blots in Fig. 1e, nuclear extracts were used. Two million cells were resuspended in 200 µL ice cold swelling buffer (10 mM HEPES pH 7.9, 10 mM KCl, 0.1 mM EDTA, 0.1 mM EGTA, 1 mM DTT, 0.5 mM PMSF) and allowed to swell on ice for 15 minutes. Cell membranes were disrupted with 12.5 µL 10% NP-40 and nuclei were pelleted. Nuclei were resuspended in ice cold buffer (10 mM HEPES pH 7.9, 0.4 M NaCl, 1 mM EDTA, 1 mM EGTA, 1 mM DTT, 1 mM PMSF). The lysate was cleared by centrifugation for 5 minutes at 19,000×*g* and the supernatant was used for blotting. Lysates were quantified using the standard BCA assay, denatured under reducing conditions in LDS buffer and 3% BME, and run on a 4–12% Bis-Tris gel. Proteins were transferred onto PVDF membranes and detected with the following antibodies: rabbit polyclonal anti-ATRX (Santa Cruz, H-300, 1:500 dilution), rabbit polyclonal anti-DAXX (Sigma-Aldrich, HPA001906, 1:1000 dilution), mouse monoclonal anti-β-actin (Santa Cruz, C4, 1:1000 dilution), rabbit polyclonal anti-KIFC3 (Sigma-Aldrich, HPA021240, 1:1000 dilution), rabbit polyclonal anti-PARP (Cell Signaling #9542, 1:1000 dilution), rabbit polyclonal anti-HA (Santa Cruz sc-805, 1:2000 dilution), anti-mouse IgG HRP (Cell Signaling 7076P2, 1:5000 dilution), anti-rabbit IgG HRP (GE Healthcare NA934, 1:2000 dilution).

### RNA-Seq

Total RNA (5 ug) was subjected to two rounds of selection using the Dynabeads mRNA DIRECT Micro Kit (Cat. No. 61021, Ambion), following the manufacturer’s instructions. The full yield of poly(A) mRNA was sheared in a Covaris S2 acoustic sonicator (60 sec, Duty Cycle = 5%, Intensity = 5, Cycles/Burst = 1000) and used in preparation of conventional TruSeq RNA libraries (FC-122-1001, Illumina). These were sequenced on an Illumina NextSeq. 500. The raw sequence data were aligned to hg19 with the STAR aligner (version 2.4.1d,). Read counts were computed with featureCounts in the Subread package (version 1.4.6). Variance stabilizing transformation (VST) and normalization in the DESeq. 2 Bioconductor package were applied to the count data. RNA and DNA sequencing data are available at dbGAP under accession number phs001495.

### DAXX siRNA knock-down

For DAXX siRNA knock-down, G292 cells were transfected with 10 pmol siRNA per well of a 6-well plate using RNAiMAX reagent. For DAXX, siRNAs used were ThermoFisher Silencer® Select siRNAs s3936 and s3937. Knock-down was normalized to transfection with Qiagen AllStars Negative Control siRNA. Knock-down was assessed at 72 hours after transfection.

### Reintroduction of DAXX in G292 cells

The pLX304 Gateway lentiviral expression vector containing DAXX cDNA from the DNASU plasmid repository (Plasmid ID HsCD00436646) was used for stable expression of wild-type DAXX. Lentivirus was produced in HEK293-T cells using the psPAX and pMD2.G packaging vectors obtained from Addgene plasmid repository (Plasmid #12260 and #12259, respectively). Target cells were infected with equivalent multiplicities of infection and selected using 15 μg/mL blasticidin. Clonal cell lines were established using single cell cloning and maintained in 2 μg/mL blasticidin.

For inducible DAXX expression, constructs were generated using Gateway cloning into the pINDUCER-20 expression vector (Addgene Plasmid #44012). DAXX cDNA was PCR amplified from the above described pLX304 vector. Primers used for Gateway cloning were: DAXX-forward GGGGACAAGTTTGTACAAAAAAGCAGGCTGGCACCATGGCCACCGCTAAC, DAXX-reverse GGGGACCACTTTGTACAAGAAAGCTGGGTCATCAGAGTCTGAGAGCACGAT, DAXX-short GGGGACCACTTTGTACAAGAAAGCTGGGTCCTTGCAAGTACCAGGCCG and DAXX-KIFC3 GGGGACCACTTTGTACAAGAAAGCTGGGTTGGCCGAGGGCTGCAGCTTCC.

For comparison of wild-type DAXX, truncated DAXX and DAXX-KIFC3 localization, constructs were transiently transfected using Lipofectamine 3000. Approximately 18 hours after transfection, cells were treated with 10 ng/mL doxycycline to induce DAXX expression. Imaging was performed after 24 hours of DAXX expression.

For generation of G292-iDAXX, lentivirus was produced in HEK293-T cells using the psPAX and pMD2.G packaging vectors. Target cells infected with equivalent multiplicities of infection and selected using 500 ug/mL G418. Clonal cell lines were established using single cell cloning and maintained in 100 μg/mL G418. For all G292-iDAXX induction experiments, cells were treated with 10 ng/mL doxycycline to induce DAXX expression for the indicated time periods.

### Immunoprecipitation

Cell lysates were prepared with IP lysis buffer (25 mM Tris HCl pH 7.4, 150 mM NaCl, 1% NP-40, 1 mM EDTA, 5% glycerol). Buffer was supplemented with protease inhibitor cocktail (HALT, ThermoFisher). Lysates were sonicated and cleared by centrifugation. Lysates were incubated with 1 µg of antibody overnight and protein complexes precipitated using Protein A/G magnetic beads (ThermoFisher Scientific). Washes were performed using TBS buffer with 0.05% Tween-20. Proteins were eluted from beads by boiling with LDS sample buffer. Proteins were analyzed by western blot as described and probed using Veriblot secondary antibody (Abcam).

### FACS cell cycle analysis

Cell cycle analysis was performed using propidium iodide staining per standard protocols. Cells were counted on a BD FACSCanto II and data were fit using FlowJo.

### Terminal restriction fragment analysis

TRF analysis was performed based on the method described previously^39^ with the following modifications. Cells were embedded in 100 µL 1% agarose plugs, digested overnight with Proteinase K, then exchanged into CutSmart buffer (NEB) and digested overnight with RsaI and HinfI. DNA was separated via pulse-field electrophoresis, using a 1% agarose gel, in 0.5xTBE. The gel was run at 6 V cm^−1^ for 15 h with switch times of 0.1–6.0 s. After separation, the gel was blotted to a positively charged membrane, which was probed with telomere specific 3′ labeled DIG probes (CCCTAA)_3_ (IDT) used at a concentration of 15 ng/mL. Detection was accomplished using the TeloTAGGG Telomere Length Assay Kit (Sigma-Aldrich).

## Supplementary information

Supplementary Information

## Data Availability

RNA sequencing data are available at dbGAP under accession number phs001495.v2.p1.
