# Supplementary material for: Rapid and reversible suppression of ALT by DAXX in osteosarcoma cells
Source: Sci Rep. 2019 Mar 14;9:4544. doi: 10.1038/s41598-019-41058-8 (PMC6418139; doi:10.1038/s41598-019-41058-8)
Supplement: Supplementary file 1 — Supplementary Information [file 41598_2019_41058_MOESM1_ESM.pdf]

Supplementary Information

Rapid and reversible suppression of ALT by DAXX in osteosarcoma cells

Kathryn E. Yost[1,2,#], Sarah F. Clatterbuck Soper[1,#], Robert L. Walker[1], Marbin A. Pineda[1], Yuelin J. Zhu[1], Corbin D. Ester[1,3], Soyeon Showman[1], Anna V. Roschke[1], Joshua J. Waterfall[1,4*], and Paul S. Meltzer[1*]

[1] Genetics Branch, Center for Cancer Research, National Cancer Institute, National Institutes of Health, Bethesda, MD 20892, USA.
[2] Current address: Center for Personal Dynamic Regulomes, Stanford University School of Medicine, Stanford, CA 94305, USA.
[3] Current address: Harvard Medical School, Boston MA 02115, USA
[4] Current address: Translational Research Department & INSERM U830, Institut Curie, Paris, France.
[#] These authors contributed equally.
[*] To whom correspondence should be addressed. Email: pmeltzer@mail.nih.gov and joshua.waterfall@curie.fr

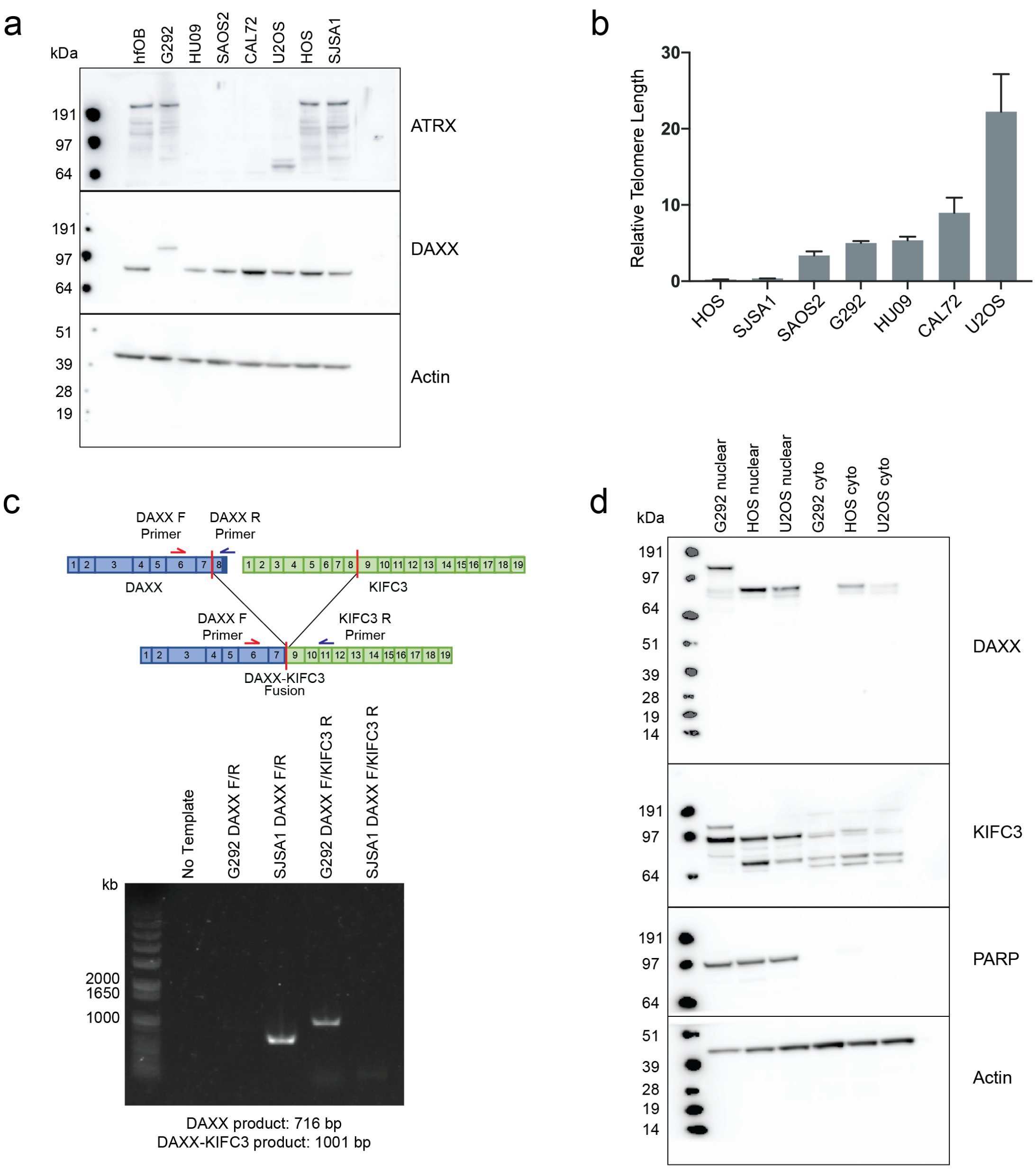

Supplementary Figure S1. Characterization of the DAXX-KIFC3 fusion, Related to Figure 1. **(a)** Extended images of blots shown in Fig. 1c. **(b)** Relative telomere length quantitative PCR results for a panel of ALT+ and TERT+ osteosarcoma cell lines. **(c)** DNA gel of RT-PCR products for DAXX and DAXX-KIFC3 transcripts showing lack of full-length DAXX transcript and presence of DAXX-KIFC3 transcript in G292. **(d)** Extended images of blots shown in Fig. 1e.

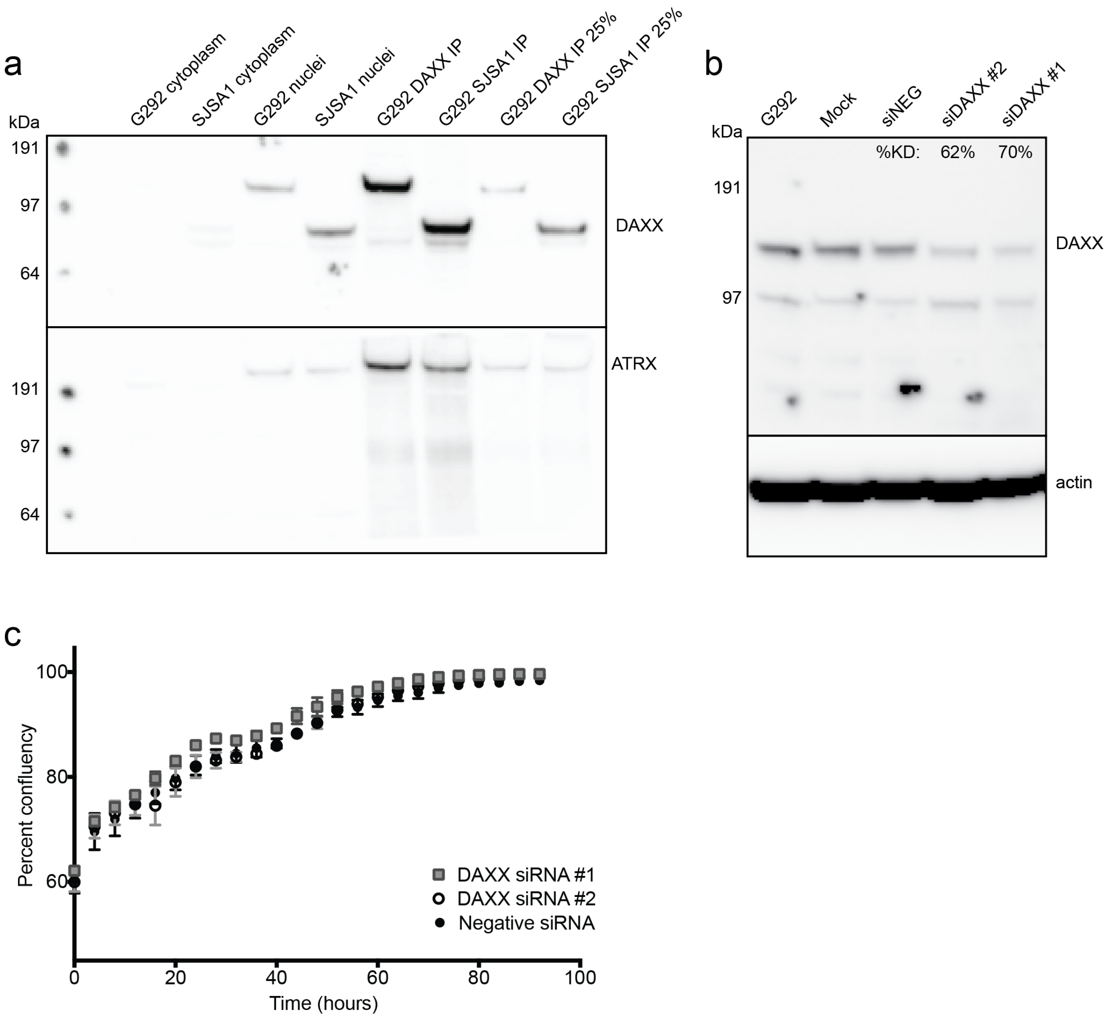

Supplementary Figure S2. DAXX-KIFC3 interacts with ATRX and is not required for proliferation, Related to Figure 2. **(a)** Extended images of blots shown in Fig. 2a. **(b)** Immunoblot showing siRNA knock-down of DAXX-KIFC3 expression in G292. Actin signal used for normalization was taken from a duplicate gel run in parallel. Due to poor knockdown, siDAXX #3 was not used for further experiments. **(c)** Growth curve showing no impact of DAXX siRNA KD on cell growth in G292.

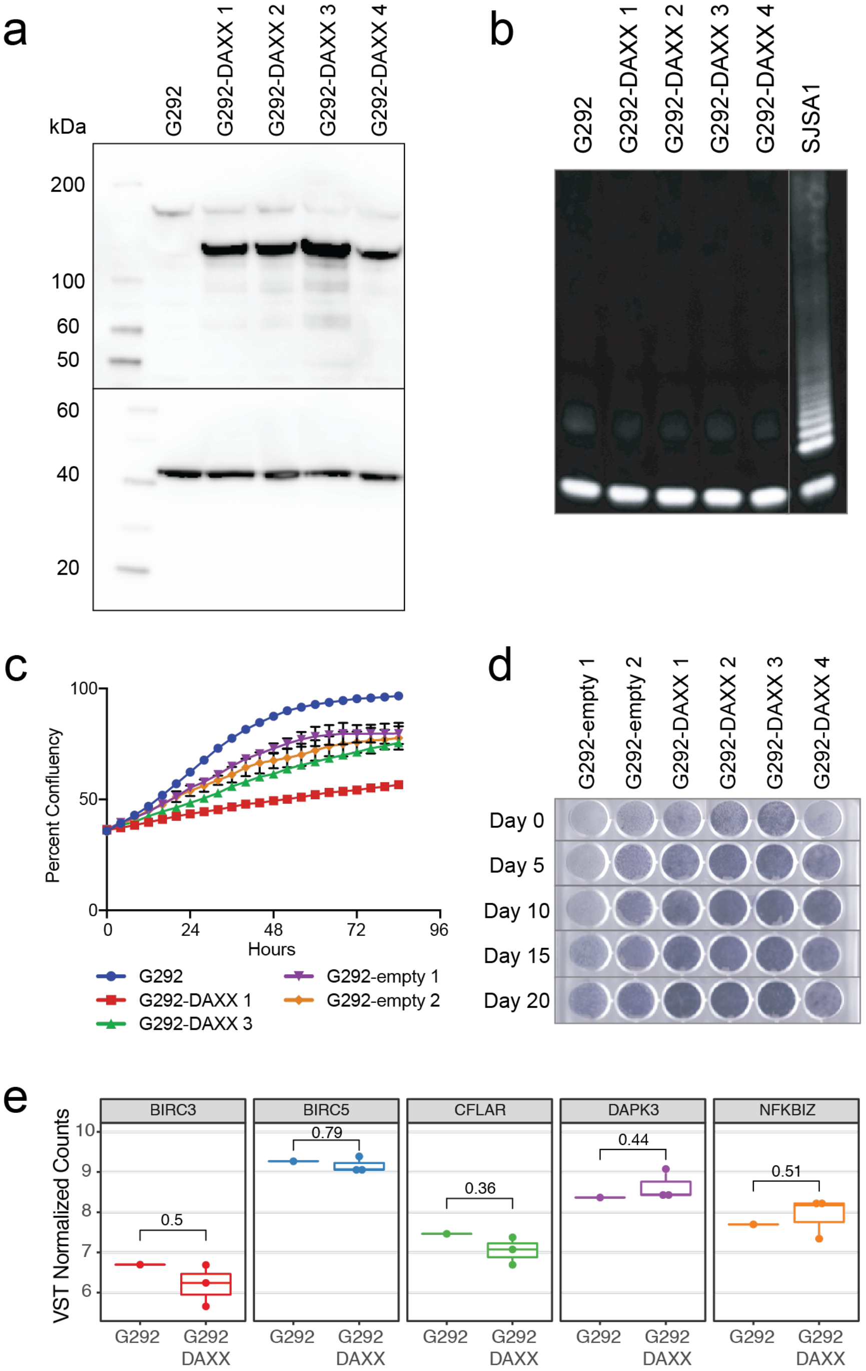

Supplementary Figure S3. Characterization of G292-DAXX cell lines, Related to Figure 3. **(a)** Extended images of blots shown in Fig. 3a showing additional G292-DAXX clones. **(b)** DNA gel of TRAP assay products showing lack of telomerase activity in G292-DAXX. All lanes are from the same gel, with non-relevant lanes between G292-DAXX clones and SJSA1 removed for clarity. **(c)** Growth curves of parental G292, G292-empty and G292-DAXX cell lines showing decreased proliferation of G292-DAXX. **(d)** Alkaline phosphatase staining following growth in osteoblast differentiation media for the indicated periods showing increased differentiation of G292-DAXX. **(e)** Comparison of normalized expression of DAXX target genes between G292 and G292-DAXX clones. Non-significant p-values shown above (Wald significance test).

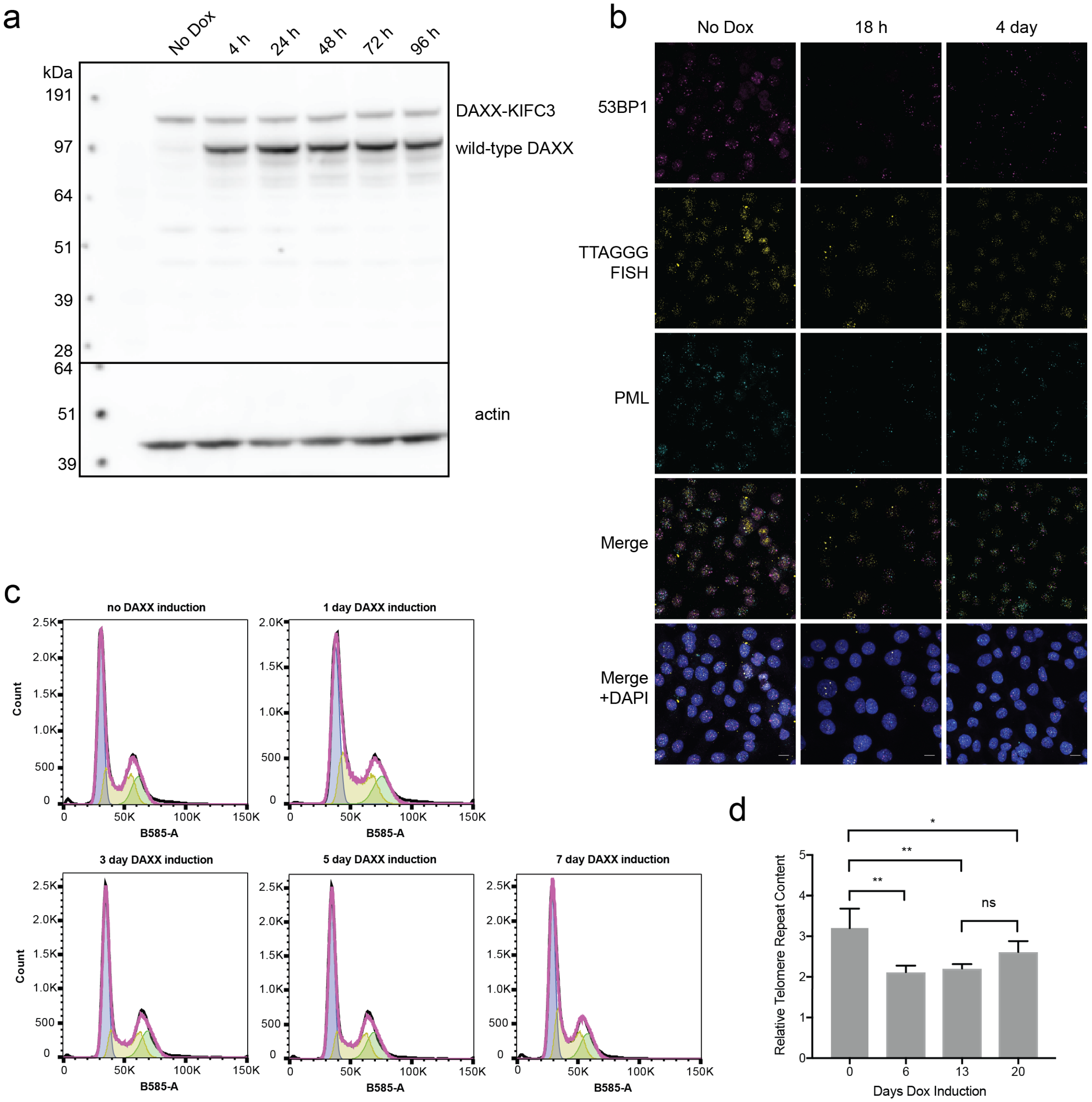

Supplementary Figure S4. Characterization of G292-iDAXX, Related to Figure 4. **(a)** Extended images of blots shown in Fig. 4a. **(b)** Sample images used for APB and TIF quantitation show in Fig. 4c,f. **(c)** Sample traces used in FACS cell cycle analysis. **(d)** Relative telomere content assay showing rapid decrease in telomere repeat content after induction of DAXX expression (ANOVA multiple comparisons test, ns: $p > 0.05$, *: $p < 0.05$, **: $p < 0.01$).

Supplementary Methods

Telomere Repeat Content Assay

Relative telomere lengths were assessed using a monochrome multiplex qPCR assay described previously[1]. Briefly, genomic DNA was extracted and quantified as stated above. Telomere monochrome multiplex qPCR was performed on 12 ng of genomic DNA, assayed in triplicate. qPCR reactions contained 1X Qiagen Quantitect Muliplex NoRox PCR master mix, 2 uM SYTO 82 nucleic acid stain and 9 uM telomere and albumin (single copy reference gene) primers. Thermal cycling and analysis were performed as described.

Growth curve

G292 growth was assayed using an Incucyte Live cell Analysis system. Confluence was assessed over four fields per well. Curves represent an average of three wells.

TRAP Assay

A non-radioactive telomeric repeat amplification (TRAP) protocol using SYBR Green I for detection of amplification products was carried out as described[2]. Cell lysates were prepared in CHAPS buffer and concentration determined using the Bradford assay. Ten micrograms of protein was added to a 25 µL TRAP reaction. Primer amounts used were 150 ng TS primer, 300 ng of ACX and NT primers and 0.7 fg of TSNT primer. Eight microliters of amplification products were run on a 10% TBE gel, stained with SYBR Green I and imaged using the LAS-3000 system (FujiFilm).

Alkaline phosphatase staining

Cells were plated on 24-well plates and allowed to grow to confluency. Differentiation was induced using osteoblast mineralization medium (PromoCell) for the designated time periods, with media changed every third day. Alkaline phosphatase activity was detected at the end of the time course. Cells were washed with DPBS + 0.05% Tween-20, fixed for one minute with 10% formalin and again washed with DPBS + 0.05% Tween-20. Cells were stained for 10 minutes at room temperature in the dark with BCIP/NBT substrate (Sigma Aldrich) before imaging.

Supplementary References

1 Cawthon, R. M. Telomere measurement by quantitative PCR. *Nucleic Acids Res* **30**, e47-e47 (2002).

2 Banerjee, P. P. & Jagadeesh, S. Non-radioactive assay methods for the assessment of telomerase activity and telomere length. *Methods Mol Biol* **523**, 383-394, doi:10.1007/978-1-59745-190-1_25 (2009).
